# Handheld Magnetic-Compliant Gamma-Ray Spectrometer for Environmental Monitoring and Scrap Metal Screening †

**DOI:** 10.3390/s22041412

**Published:** 2022-02-12

**Authors:** Marco Carminati, Davide Di Vita, Giuseppe Morandi, Ilenia D’Adda, Carlo Fiorini

**Affiliations:** 1 Dipartimento di Elettronica, Informazione e Bioingegneria, Politecnico di Milano, 20133 Milano, Italy; davide.divita@polimi.it (D.D.V.); ilenia.dadda@polimi.it (I.D.); carlo.fiorini@polimi.it (C.F.); 2 Istituto Nazionale di Fisica Nucleare (INFN), Sezione di Milano, 20133 Milano, Italy; 3 TNE S.p.A.—Technology Nuclear Electronics, Cassina de’ Pecchi, 20060 Milano, Italy; giuseppe.morandi@tnenuclear.com

**Keywords:** electromagnet, metal recycling, SiPM, radioactivity monitoring, wireless sensor

## Abstract

Spotting radioactive material in waste is of paramount importance for environment protection. This is particularly challenging when orphan sources are hidden in scrap metal that shields their activity from the traditional detectors in the portals scanning incoming trucks. In order to address this issue, we present a wireless and compact SiPM-based gamma spectrometer compatible with strong magnetic fields (0.1 T) to be installed in the bore of the lifting electromagnets to scan reduced volumes of metal and thus achieve higher sensitivity. The microcontroller-based instrument provides 11% energy resolution (at 662 keV), an energy range from 60 keV to 1.5 MeV, a max. count rate of 30 kcps, a weight <1 kg, and a power consumption <1 W. The results of its extensive characterization in the laboratory and its validation in the field, including operation in a scrap yard as well as on a drone, are reported.

## 1. Introduction

Silicon photomultiplers (SiPM) are solid-state photodetectors endowed with internal gain, composed of arrays of single-photon avalanche photodiode cells connected in parallel. They combine single-photon sensitivity with a wide dynamic range of the analog output signal [1]. Their application has been quickly expanding in several areas from LiDAR and time-of-flight sensing to nuclear spectroscopy and medical imaging, where they are coupled to scintillators, and many other low-light applications such as dark matter physics. Despite a slightly lower quantum efficiency and higher dark counts, they have been replacing photon-multiplying tubes (PMT), traditionally employed in gamma-ray sensing [2], thanks to various advantages such as: (1) miniaturization and robustness (mm-sized silicon chips as opposed to glass tubes of ~10 cm diameter and length), (2) lower operating voltages (tens of volts as opposed to several hundreds), (3) higher gain and (4) extremely low sensitivity to magnetic fields.

The compatibility of SiPMs with strong magnetic fields (up to a few Teslas) has been a key factor enabling the growth of multimodal medical imaging, in particular allowing the realization of gamma cameras for PET [3] and SPECT [4] scanners compatible with MRI, i.e., that can be operated inside the bore of a MRI scanner during imaging sequences.

Motivated by the success of MRI-compatible gamma imagers, we have conceived an industrial spin-off application of this technology in the area of waste and recyclable material monitoring. In particular, a very critical case concerns the presence of radioactive sources in scrap metal. Radioactive sources are largely used in industrial and medical applications, and they can be accidentally or intentionally disposed in waste. The fusion of a radioactive material among recyclable metal in a steel mill would create a severe environmental accident by contaminating the whole plant and semifinished products [5]. For this reason, metal-processing plants are mandatorily required by national environmental regulations to scan the incoming material. Portals equipped with large plastic scintillators are installed at the entrance gates for both trucks and train wagons accessing the scrap yard [6]. However, due to the large amount of metal present in these containers of large volumes (50–100 m^3^), radioactive source can be heavily shielded. Thus, it is possible that fixed monitors miss the presence of dangerous items. In order to address this issue, we propose to install a radioactivity detector in the electromagnet used to lift and manipulate the scrap metal in the yard, for instance, during the loading in the hopper for fusion. This would allow for increasing the sensitivity in the detection of orphan sources, thanks to the smaller volume of metal (max. ~1 m^3^) lifted in the cluster under the magnet.

**Figure 1:**
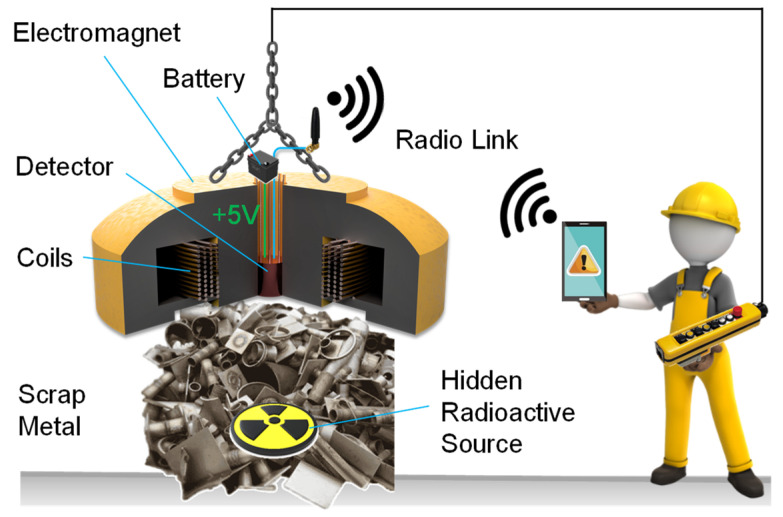
Schematic representation of the gamma-ray spectrometer here presented. Its main application is safe identification of radioactive material hidden in scrap metal with enhanced sensitivity thanks to the installation of the detector in the bore of the lifting electromagnet operated in the scrap yard prior to fusion.

In this paper we present the design and experimental characterization of a wireless, compact and robust gamma-ray spectrometer compatible with large magnetic fields and, thus, designed to be installed inside lifting electromagnets for scanning scrap metal in scrap yards prior to fusion in steel mills (Figure 1). In the case of detection of a radionuclide, an alarm is immediately transmitted to the magnet operator to prevent contamination of the plant. This unit can be both installed in new magnets as well as retrofitted into already existing magnets, by drilling a hole (<25 cm) in the middle of the bore.

The paper is organized as follows: Section 2 illustrates the design of the instrument, including the scintillator choice, the optimization of SiPM arrangement and the electronic and mechanical design. Section 3 reports the results of the experimental characterization, both in the laboratory and in the field, while conclusions are drawn in Section 4.

## 2. Instrument Design

### 2.1. Design Specifications

The increase in sensitivity expected from embedding the detector inside the lifting electromagnet is achieved at the cost of extremely challenging operating conditions for the spectrometer, namely: (1) large magnetic fields (up to a tenth of a Tesla), (2) high temperature (up to ~70 °C in the bore of the magnet), (3) intense vibrations and mechanical shocks (since the magnet is also used as a hammer to dig and compact scrap metal in the yard) and (4) short measurement time (a few seconds during the maneuver of the lifted metal cluster).

The sensor should nevertheless offer proper sensitivity and energy resolution (~10% Full-Width Half-Maximum (FWHM) at 662 keV) to identify the different radionuclides [7]. Another challenge is the variability of the background, since the instrument will be quickly moving to different areas in the scrap yard. Finally, it should be also affordable in order to be installed in several stations and machines. The energy range to be covered is from 60 keV up to 1.5 MeV to address most artificial sources and be in line with commercial instruments. The most common isotopes, with long decay times, that are searched for in scrap metal are ^137^Cs (662 keV) [8] and ^60^Co (1.17 and 1.33 MeV) [9], since they are extensively used in the form of sealed gamma-ray sources in industrial applications, such as sterilization of food and, along with ^192^Ir and ^75^Se, tomography, in particular of high density materials, as in metallurgy and welding verification. Another relevant isotope is ^131^I (364 keV), which is used in nuclear medicine and is also monitored in air samples collected in the atmosphere (typically by flowing large volumes of air on filters). In fact, when released from reactors, for instance, due to nuclear accidents, it partially binds to aerosol, similarly to ^137^Cs, and its concentration in air is routinely monitored by networks of ground stations managed by environmental protection agencies of several countries [10]. A maximum count rate of at least 10 kcps should be granted by the instrument.

### 2.2. Scintillator and SiPM

Given the wide energy range to be covered, an indirect conversion architecture was selected, thanks to its versatility. A scintillator converts the incoming gamma photon into a handful of optical photons collected by SiPMs coupled to one face of the crystal. The best compromise between detection properties and cost is represented by the thallium-doped sodium iodide NaI(Tl) scintillator, characterized by a material density of 3.6 g/cm^3^, a light yield of 38 photons/keV, leading to an excellent intrinsic energy resolution of ~6% [11], an emission wavelength at 415 nm (matching PMT and SiPM responsivity) and a decay time τ below 250 ns, thus being potentially suitable for large count rates (though not common in environmental monitoring applications). Furthermore, this scintillator is suitable for low-background applications, including environmental ones [12]. The only weak aspects of this scintillator are related to its long-term aging: in fact, it is hygroscopic (and thus is typically sealed in thin metal cases with a quartz window for optical coupling with the photodetectors), and it is sensitive to temperature gradients (max. ~10 °C/hour).

For a gamma photon of 1 MeV energy, the linear attenuation coefficient in NaI is ~0.2 cm^−1^, requiring a thickness of ~1 cm for a complete absorption. At the same time, the volume of the scintillator cannot be too small in order to collect enough events for statistical relevance. In fact, the standard size of NaI scintillators for portable gamma spectrometers for environmental monitoring is 2″ [13]. The final choice related to the scintillator concerns its shape and the number and arrangement of SiPMs attached to its face. Since individual SiPMs are currently still relatively expensive (~$100 per piece for small batches), an array of a few SiPMs easily matches the cost of the scintillator, the most expensive component of the spectrometer. The identification of the optimal trade-off between the number of SiPMs (i.e., cost) and the system spectroscopic performance is crucial for the commercial success of this development.

**Figure 2:**
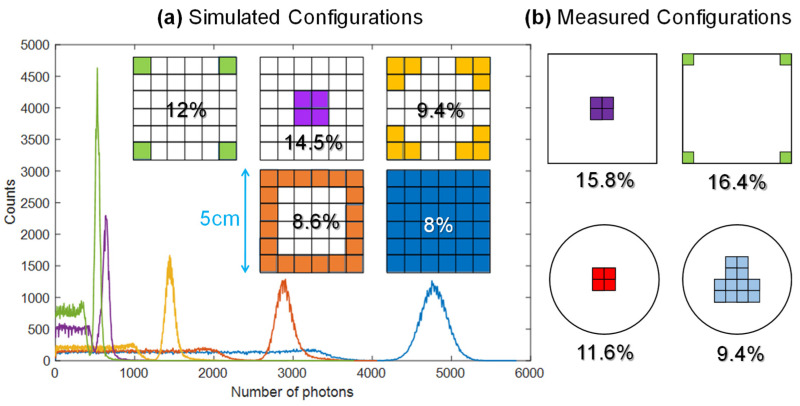
Analysis of the optimal SiPM layout for both cubic and cylindrical NaI scintillators coupled to square 6 mm × 6 mm SiPMs: FWHM energy resolution at 662 keV is reported (**a**) by means of simulations performed with the ANTS2 software [15] and (**b**) confirmed by experimental measurements on both square and cylindrical geometries.

The starting point for the analysis was a paper by Saint Gobain [14] suggesting that acceptable spectroscopic performance could be achieved by placing only 4 SiPM at the corners of a cubic scintillator, whose surface is covered by a reflective layer. The reason is that the corners act as light guides, concentrating most the scintillation light along them. We have performed simulations to validate this concept with the ANTS2 software package [15], considering square SiPMs of 6 mm side and a cubic NaI scintillator of 5 cm side. As reported in Figure 2, simulations confirmed this promising approach: the four-corner layout (green) achieved an energy resolution at 662 keV of 12% FWHM as compared to the best case of 8% achieved by a full coverage of the bottom face (blue). Thus, we built a first prototype of detector with a cubic scintillator and 4 SiPMs [16] and measured its energy resolution, which turned out to be significantly worse (16.4%). Since electronic noise is negligible at these energies [17], the reason for this discrepancy is ascribed to the lateral loss of light in the optical interface between the scintillator and the SiPM, which in simulations is considered ideal, while, in the real case, some light is escaping from this optical layer, especially in our demonstrator, where this delicate coupling was manually assembled (either with optical grease or a silicone pad).

Other configurations were then measured and simulated, as summarized in Figure 2 and Table 1: placing 4 SiPMs in the middle of the cubic crystal (purple) offered slightly better resolution (15.8%), while a significant improvement was achieved by moving to a cylindrical scintillator. In fact, by using 4 SiPMs in the middle (red), a resolution of 11.6% is achieved. It can be improved to 9.4%, in line with state-of-the-art devices for similar applications [7], by mounting 12 SiPMs in the middle in a “T” arrangement (cyan).

In conclusion, based on this study we opted for a cylindrical scintillator of 2″ diameter and thickness coupled to an array of 4 SiPM placed in the middle and surrounded by a reflective layer. Although a 2″ cylindrical scintillator has 30% less volume than the cubic equivalent, its shape is more standard and easily machinable, making it less expensive, too.

### 2.3. Electronics

**Figure 3:**
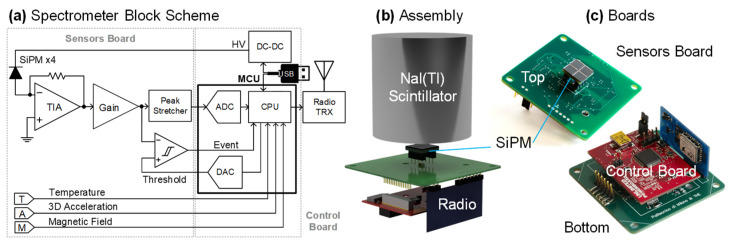
Instrument electronics: (**a**) schematic, (**b**) final assembly and (**c**) view of the PCBs.

The block scheme of the electronic circuit is shown in Figure 3. Since only spectroscopy is performed by this instrument, the currents from all the SiPMs are merged into a single current input, the virtual ground of a transimpedance amplifier (TIA), to obtain a single larger pixel [18]. The TIA is followed by a gain stage, a comparator with a selectable threshold and a peak stretcher, storing the peak amplitude of each event. A 32-bit microcontroller of the STM32 family manages the operation of the instrument. Its internal ADC samples the output of the peak stretcher, triggered by each detected event. One internal DAC is used to set the detection threshold and a second one to drive a DC-DC converter generating the high voltage (HV), biasing the SiPM. Finally, the microcontroller handles the communication with USB (through an FTDI bridge) and radio communication.

Additional sensors were included in the circuit: a temperature sensor (a PT100 thermistor) to measure the temperature of the SiPM and thus stabilize its gain in closed-loop by adjusting the DC-DC HV [19], a tri-axial MEMS accelerometer (with SPI interface, data rate from 0.5 Hz to 1 kHz and selectable full-scale range of ±2 g, ±4 g and ±8 g) to detect the motion of the unit and a solid-state magnetometer (a Hall-effect CMOS sensor with 10 µs response time, ±176 mT full-scale range and 7.5 mV/mT sensitivity) to detect the status of the electromagnet, corresponding to the presence of the lifted metal cluster.

The value of the feedback resistor of the TIA has to be set depending on the number of SiPM. The SiPM peak current of each pulse, typically in the mA range, is given by the total collected charge divided by a signal time constant τ_s_ given by the convolution of the scintillator decay time τ and the SiPM microcell recharge time constant (~50 ns). The charge is given by the number of photoelectrons impinging on the SiPM multiplied by the collection efficiency, the photo-detection efficiency (PDE) and the gain (~10^6^, depending on the biasing overvoltage) of the SiPM. For simplicity, a simple RC shaping is applied to the filter the pulse by means of the TIA time constant. Here, the filter is set at 1.4 µs, consistent with 5·τ_s_. Furthermore, the stability of the TIA has to be carefully studied, given the large capacitance of the SiPM (~1 nF per pixel). For a large number of SiPM connected to a single current input, the stability of negative feedback can become critical, and other approaches, such as approaches based on weakly positive feedback, can be adopted [20].

After trying different devices, a compact commercial transceiver module was selected for the radio link. It operates in the free band of 900 MHz with FSK modulation, +9.5 dBm output power (40 mA max. current consumption), a RF data rate of 153.6 kbit/s and UART interface at 115,200 bit/s. A range above 1 km in free space of the radio link was experimentally verified: it is suitable for operation in the scrap yard, even in the presence of shielding structures.

The circuit is realized in three separate printed circuit boards, visible in Figure 3. The sensors board (with a green solder mask, it is the largest, at 58 mm × 58 mm) hosts the SiPMs (on the top layer) and all other sensors and the front-end circuits (on the bottom). It also includes the connector for the radio module that is plugged in vertically (blue solder mask), while the control board (red solder mask) hosts the microcontroller, the USB interface, power regulators and the DC-DC converter. The choice of components and the layout of the PCBs followed the indications for compatibility with intense varying magnetic fields developed for SPECT/MRI [4].

The system is powered by a single +5 V supply, provided by a laptop through the USB interface or by a battery, which will be located in a rugged box on top of the electromagnet, together with the antenna. The instrument current consumption is 130 mA during acquisition mode, and it reaches a max. of 180 mA during radio transmission for an average power consumption well below 1 W.

### 2.4. Mechanics

**Figure 4:**
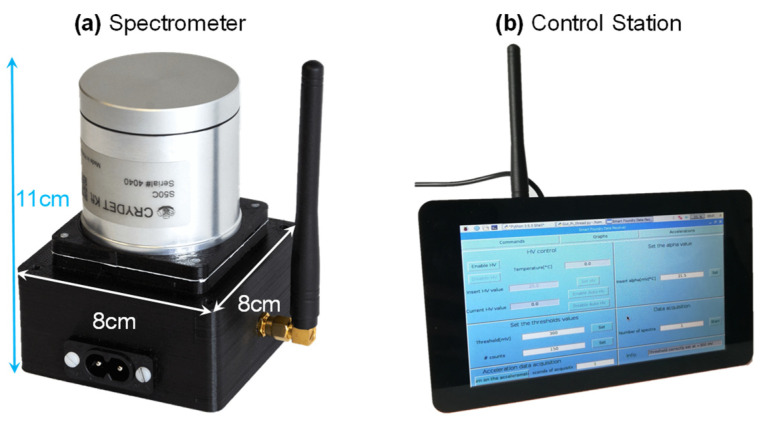
Photographs of the realized prototypes: (**a**) spectrometer (here without the lid of the very compact plastic enclosure for handheld use) and (**b**) wireless stand-alone control station consisting of a Raspberry Pi board and touchscreen.

When used in the laboratory or as handheld portable spectrometer, a simple 3D-printed black PLA case is used to close and protect the instrument, providing light-tightness (Figure 4). The optical coupling between the SiPM and the output surface of the scintillator is critical in terms of spectroscopic performance. For portable use, an optical grease (BC-630 by Saint Gobain) is commonly used, while for industrialization and installation in harsh environments such as the electromagnet, a silicone optical pad (EJ-650 by Eljen Technology) is used. Beyond granting optical coupling, it also acts as a damper of vibrations.

Since the NaI crystal is prone to cracking and cleaving, degrading energy resolution, due to mechanical stress, a special enclosure was designed and realized for installation of the instrument in the bore of the electromagnet. It consists of two custom-designed co-axial steel cylindrical cases. The internal one (~10 cm diameter) has a similar volume to the plastic one and keeps the electro-optical assembly tightened and light-proof. The external one (~20 cm diameter) surrounds the detector, providing mechanical protection, thermal isolation and mechanical dampening. In fact, a set of shock absorbers made of Teflon and steel springs connects the internal case with the external one, granting mechanical decoupling. We have estimated that the highest acceleration suffered by the unit would be ~17 g in the case of collision with a rigid wall of the magnet (of max. 10,000 kg mass) at a max. speed of 1.3 m/s and assuming a spring compression of 10 mm. This value decreases significantly to a few g in the realistic case of impact of the magnet with scrap metal that compressed by more than 30%, dissipating the energy of the collision. Preliminary laboratory tests with accelerations of a few g were performed, and no damage to the crystal was observed. In any case, in case severe collisions are expected, a less delicate crystal such as CsI could be taken into consideration to replace NaI, trading off energy resolution with mechanical robustness. A bottom steel lid with holes, despite reducing the sensitivity, can be mounted to protect the spectrometer from damage due to sharp metal rods.

### 2.5. Embedded Processing and Software

In addition to managing the communication with peripherals and the acquisition of the sensors signals, the microcontroller performs a major embedded processing task: building the spectra in real time. Conversion from ADC bins to energy values is performed by a linear map identified by means of calibration. If the energy of each detected event falls within an acceptance window, it is counted and the bin corresponding to its energy is increased. Despite the ADC has a resolution of 12 bits, spectra are re-binned to 10 bit (i.e., with ~1.3 keV/bin) by merging bins, to be compatible with the majority of standard analytical software for gamma monitors. The minimum acquisition time of gamma events is currently set to 1 s. All auxiliary sensors are also sampled every second. The user can set a total measurement time and receive the final integrated spectrum or opt to receive partial spectra every second. The acquisition time of each event (equal to ~10 µs and dominated by the conversion time of the internal ADC) is taken into account as dead time, in particular at high rates, to measure the effective active measurement time. In this configuration, a maximum count rate of 30 kcps can be achieved. This value is suitable for environmental monitoring. However, in case a larger count rate capability would be needed, the ADC could be easily replaced with an external one offering a faster conversion time. Two auxiliary processing steps are the stabilization of the SiPM gain versus temperature and the correction of the baseline shift for high count rates, described in Section 3.1.2.

As anticipated, the spectrometer can be operated in two modes: when connected to a PC, spectra are streamed via USB port that provides also power supply. Instead, when battery powered, it operates as a standalone unit and communicates via radio. A graphical user interface (GUI) was developed to control the instrument. It allows setting all acquisition parameters and displaying acquired spectra and the values of auxiliary sensors. A first version of the software was written in Matlab^TM^, and it is still used when operating via USB connection. Then, it was migrated to Python in order to run in Linux on a Raspberry Pi B+ single-board microcomputer connected to a twin radio transceiver and coupled with a touchscreen, thus realizing a portable standalone receiving station (visible in Figure 4) for demonstration purposes.

### 2.6. Calibration and Self-Diagnostics

Energy calibration of the spectrometer is performed in the laboratory at known temperature and by means of two or three calibration sources such as ^131^Ba, ^137^Cs and ^60^Co in order to cover a wide energy range from 80 to 1330 keV. Since the dominant contribution to spectrometer stability is the thermal drift of the gain of the SiPMs, its stabilization is sufficient to grant reproducibility. A typical warm-up time of a few minutes is required.

Finally, given the safety role of the sensor, it would be important to add some degree of self-diagnostic capability to the instrument. The ideal solution, which is a standard option in more sophisticated systems [21], would be to add an LED injecting light pulses in the scintillator. In this way, the whole chain can be periodically checked, between measurements. In case of fault, the operator can be immediately alerted and the use of the instrument interrupted to avoid false negatives. Since the mounting of the LED coupled with scintillator can be critical from a mechanical point of view (both in terms of robustness and light-tightness), an alternative approach is to produce electrical pulse from the HV line, injecting test current pulses through the SiPM capacitance. However, this requires making the low-pass filters of the HV line switchable and does not account for damage to the scintillator or its coupling with the SiPMs.

## 3. Results

### 3.1. Laboratory Characterization

#### 3.1.1. Energy Resolution

**Figure 5:**
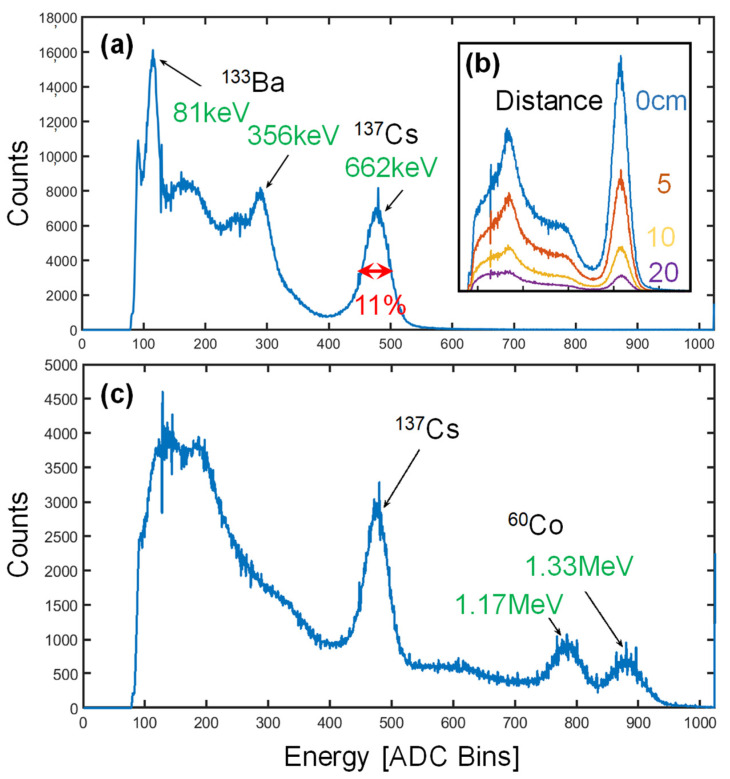
Spectroscopy of gamma sources: (**a**) barium and cesium, (**b**) at different distances from the detector and (**c**) cesium and cobalt isotopes.

The spectrometer was initially characterized in the laboratory with sealed calibration sources, as reported in Figure 5. Energy calibration was performed, exposing the unit to both ^133^Ba and ^137^Cs sources. The measured energy resolution at 662 keV is 11% (Figure 5a). As shown in the inset (Figure 5b), when changing the distance of the source from the detector (from 0 to 20 cm), the amount of counted events in the photopeak in a fixed acquisition time (60 s), scales as expected with the square of the distance. Eventually, a ^60^Co was also used to explore the high-energy region of the spectrum. The two peaks of the ^60^Co lines at 1.17 and 1.33 MeV are clearly distinguishable, with an improvement in resolution with respect to the previous prototype with a cubic scintillator [16].

#### 3.1.2. Count Rate

**Figure 6:**
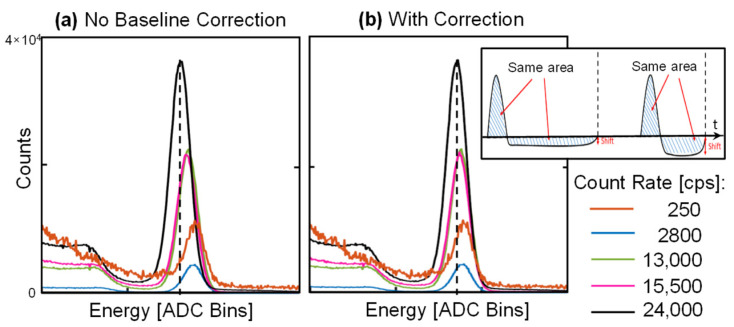
Experimental demonstration of the effectiveness of the digital correction of the baseline shift at high count rates (here generated in the laboratory with a calibration ^137^Cs source).

Due to the AC coupling between the TIA input stage and the following gain stage, for every detected event, the baseline shifts down by an area equal to the pulse area. At low count rates, the negative shift is negligible, since it is spread along a long time distance between pulses. Instead, at high rates, this effect can distort the spectrum. Since, for simplicity, no baseline holder circuit has been included, a correction of this effect in digital processing domain was introduced. The correction is applied every second: the area corresponding to the pulses in the histogram is computed and divided by the acquisition time in order to estimate the average shift. The spectrum is thus shifted to the right of such estimated amount. As reported in Figure 6 for the ^137^Cs peak at 662 keV, the correction is effective in reducing the centroid shift to less than 6 bins (negligible when compared with ~50 bins corresponding to the peak FWHM) for count rates up to 24 kcps. Of course, this simple embedded algorithm works better in the presence of a dominant photo-peak.

#### 3.1.3. High Temperature Operation

**Figure 7:**
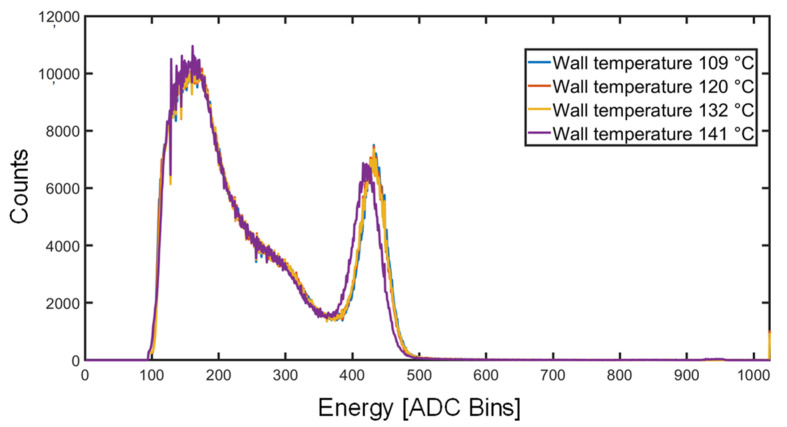
Experimental demonstration of the rejection of thermal effects when the external case temperature exceeds 100 °C and the internal one reaches ~70 °C.

Once the spectrometer was installed in the rugged metallic enclosure, it did not fit inside standard laboratory thermal chambers. Thus, the external case was wrapped with a thermal jacket containing high-power resistors and PT1000 thermistors in order to test the high-temperature behavior of the instrument. In order for the detector to reach ~70 °C, the estimated max. operating temperature, the external case was heated up to above 100 °C. The temperature was increased slowly (below 10 °C/hour) to avoid damage to the scintillator, and ^137^Cs spectra were acquired for 10 min. As visible in Figure 7, from 109 °C to 132 °C, the centroid of the photopeak does not move (it shifts of only 2 bins), demonstrating proper behavior of the stabilization loop. When increasing to 141 °C, the spectrum shows a slightly higher shift (9 bins). This effect, still negligible, is ascribed to a slight degradation of the SiPM itself, since when measured back at room temperature, it showed an irreversible slightly altered response. In fact, since, in this last measurement, the detector temperature reached ~80 °C, it was very close to the upper limit (+85 °C) of the recommended operating temperature range. In any case, this upper limit is significantly higher that the max. operating temperature expected in the magnet bore. Furthermore, if needed for a particularly hot environment, active cooling of the detector could be added.

### 3.2. Field Validation

#### 3.2.1. Inertial Test

**Figure 8:**
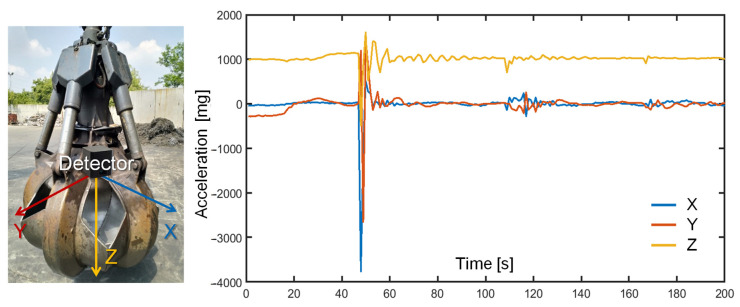
Recording of the inertial accelerations suffered by the unit mounted on a grapple operating in the scrap yard. During maneuvers, a max. of 4 g is reached.

In order to assess the intensity of the accelerations suffered by the instrument during maneuvers with scrap metal and to demonstrate its compliance with mechanical shocks, the unit was battery-powered and firmly attached to a grapple used in the scrap yard; accelerations were wirelessly recorded during realistic manipulations, as reported in Figure 8. Despite the low sampling rate of 1 Hz, insufficient to properly capture fast dynamics, it was possible to observe that the hardest maneuvers apply an acceleration reaching ~4 g in the horizontal plane and ~2 g in the vertical direction. The prototype successfully survived the stress test. The need for an accelerometer with a full-scale range larger than 4 g was confirmed. Given the smaller mass of the grapple with respect to the electromagnet, it is expected that the latter will suffer less accelerations. Measurement of the acceleration can enable the identification of the position of the sensor (at ground level vs. at the top) and the motion status (still vs. moving), which are useful to synchronize the measurement with the lifting operations, along with the magnetic sensor, and adapt the background subtraction to the different positions.

#### 3.2.2. Scrap Metal Detection

**Figure 9:**
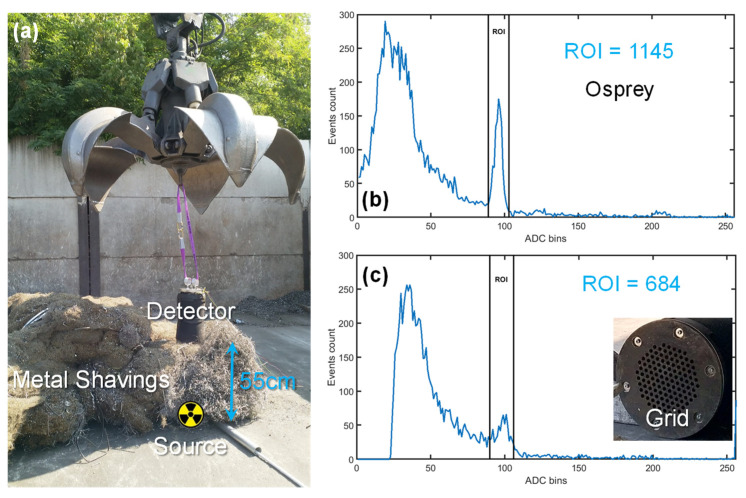
Detection of a low-activity calibration ^137^Cs source hidden underneath 55 cm of metal shavings in scrap yard (**a**), by means of a commercial spectrometer (**b**) and the proposed detector (**c**).

The spectrometer was tested in the field, in a real scrap yard. As visible in Figure 9, it was lifted by a grapple and placed on a cluster of scrap metal of 55 cm thickness, i.e., half of the max. thickness of the scrap volume (~1 m^3^) under the electromagnet. From previous tests [16], we observed that the most challenging types of scrap metal are metal shavings due to the higher density and, correspondingly, higher screening of radiation. Thus, we used metal shavings. A low-activity calibration ^137^Cs source was placed, by means of a metal pipe, underneath the cluster, and spectra were acquired. In order to have an accurate reference, a commercial gamma spectrometer Osprey^®^ (by Mirion) mounting the same 2″ NaI(Tl) scintillator coupled to a PMT and endowed with an embedded MCA (with better energy resolution but not compatible with magnetic fields) was also used and placed close to the detector under test. The reference instrument was lacking the protection grid. As shown in Figure 9, after 60 s of acquisition, both detectors could clearly identify the radioactive source. Spectra were re-binned to 8 bits for a direct comparison with the Osprey. In the region of interest (ROI) around the 662 keV photopeak (from bin 90 to 106), the Osprey counted 1145 events, while the detector under test counted 684 events. Even by considering a 1 s measurement, the ROI contains more than 10 counts that are sufficient for detection of sources of weak activity (~100 kBq). This demonstrates the capability of the proposed instrument to quickly detect the hidden source and trigger the alarm during the maneuver of the lifted scrap metal cluster. The decrease in counts with respect to the reference instrument (placed at the same distance and having the same scintillator volume) is due to the thick protection grid of our prototype (visible in the inset of Figure 9) and quantified by this comparative measurement into a loss of 40% of events.

#### 3.2.3. Operation in the Magnetic Field

**Figure 10:**
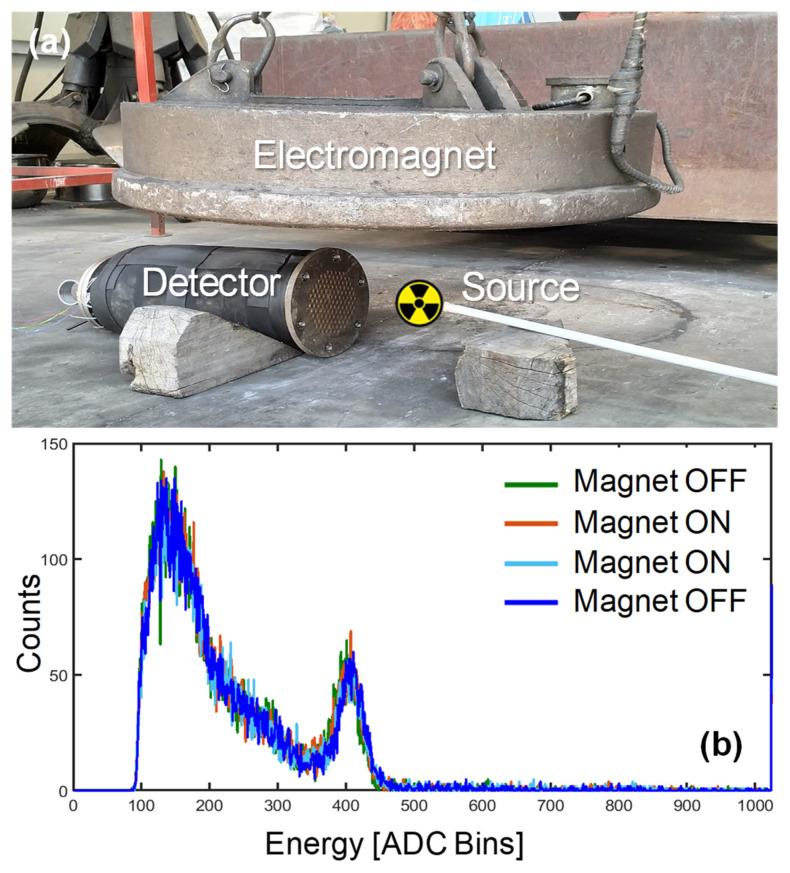
Validation of the spectrometer operation when immersed in a strong (0.1 T) magnetic field under an electromagnet used to load scrap metal: (**a**) setup, (**b**) measured spectra showing no alteration due to the field.

Finally, the compatibility with a large magnetic field was tested in the same yard by placing the detector under a lifting electromagnet. The same calibration source was placed facing the detector, and spectra were again acquired for periods of 60 s. The intensity of the magnetic field was measured to be ~0.1 T with a portable probe (Gaussmeter GM08 by Hirst Magnetics). Although the used electromagnet is a bit smaller than the ones employed in the largest scrap yards of steel mills, the intensity of the field in the final installation position, i.e., in the bore, is expected to be even lower than 0.1 T. As shown in Figure 10, there is no visible difference between the spectra measured with the magnet OFF and ON. Four spectra were measured: initially with the magnet OFF (green), then two scans with the magnet ON (red and cyan) and a final one again with the magnet turned OFF (blue). This demonstrates the correct operation of the instrument inside a strong magnetic field.

#### 3.2.4. Drone Flight

**Figure 11:**
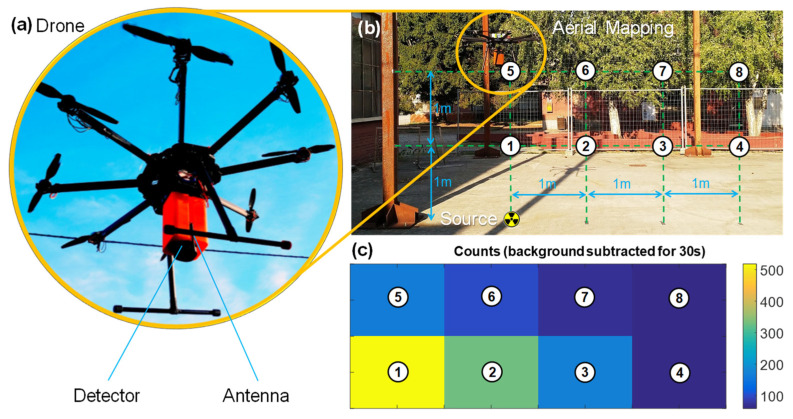
Demonstration of operation of the spectrometer embarked on a drone (**a**) for aerial mapping the activity of a ^137^Cs source placed on the ground (**b**) creating a map with 1 m spatial resolution on a vertical plane after background subtraction (**c**).

Given the compactness, low power (below 1 W) and low weight (below 1 kg in the plastic enclosure) of the unit, another test relevant for radioactivity environmental monitoring was performed. The wireless spectrometer was embarked on a drone, and the capability of aerial mapping was demonstrated. The detector was mounted by means of a 3D-printed adapter with the scintillator facing down. In order to lift a payload of 1 kg, a Tarot Iron Man 1000 Octocopter with eight propellers was employed. The instrument was powered directly from the drone battery: a standard six-cell pack with a capacity of 14,000 mAh. Since the battery generates 22 V, a switching DC–DC regulator was added to reduce the voltage to 5 V. The additional weight and power consumption of the spectrometer shortened the flight time from 30 min to 25, which is an acceptable value for land surveys. As visible in Figure 11, the drone was flown over a low-activity calibration source: it was kept hovering for 30 s in positions on a vertical square grid with pitch of 1 m. The vertical map shows the counts measured in the ^137^Cs ROI after subtraction of the background in each spot. The decrease in counts when moving away from the source follows the quadratic law, confirming the correct acquisition and showing potential for land mapping.

Several systems combining drones and gamma spectrometers have been reported [12,22] and are commercially available for this purpose (by CAEN, Mirion, Kromek etc…), but the one here proposed combines acceptable spectroscopic performance with very low weight and low power operation, in line with the state of the art achieved by means of scintillators offering higher performance such as CeBr_3_ [23].

## 4. Conclusions

We have presented a wireless gamma-ray spectrometer based on the combination of only four solid-state SiPM photodetectors and a NaI(Tl) scintillator. The main feature of the instrument is its compatibility with magnetic fields of 0.1 T, which makes it suitable for installation inside a lifting electromagnet, along with thermo-mechanical robustness and miniaturization of the electronics readout comprising an analog processing front-end and microcontroller-based embedded processing. A careful set of simulations and experimental tests allowed for optimizing materials and geometry for the best compromise between cost and energy resolution. Although the energy resolution (11%) is slightly worse than the state of the art with these components (~8%) [24], the unit is meant to serve as the ultimate safety barrier to alert and stop the manipulation of a small-volume metal cluster, to be eventually analyzed with a traditional spectrometer for better identification of the chemical species. Moreover, the same compact electronic platform could offer enhanced energy resolution by employing more SiPMs and higher-quality scintillators such as those based on cerium or LaBr_3_.

The sensor was extensively tested in multiple conditions, including operation at high temperature, high acceleration and in high magnetic field. Furthermore, thanks to the portability (<1 kg) and low-power (<1 W) of the core unit, it was also embarked on a drone.

Within the framework of the IoT paradigm, we envision potential improvements in spatio-temporal mapping of the environment, as is already happening for air and water quality [25], by the spread of networks of radioactivity monitoring stations based on units such as the one here presented, either fixed or movable, either ground-based or airborne, and operated by either humans or autonomous robots [26]. Direct involvement of the population (through paradigms such as citizen science and gamification) with low-cost smartphone-based personal detectors has also been already proposed for radioactivity [27].

Moreover, the combination of such detectors with machine learning looks extremely promising, both at single-detector level, for instance, to improve the quality of the spectrum [28] or to endow with directional sensitivity a collimator-less gamma spectrometer based on the same geometry (a cylindrical LaBr_3_ scintillator coupled to planar arrays of SiPM) [29], and at the level of merging the information captured from multiple sensing sites and fusing radioactivity maps with other heterogeneous and complementary information, such as meteorological and acoustic sensors in the case of underwater radioactivity observations [30].

## Figures and Tables

**Table 1:** Results of simulations and measurements of the energy resolution (FWHM at 662 keV) for different arrangements (Figure 2) of SiPMs (6 mm side) on a 5 cm NaI(Tl) scintillator, corresponding to different fractions of scintillator area coverage.

Crystal Shape	# SiPM	Layout	Covered Area	Simulated	Measured
Cubic	36	Full coverage (blue)	100%	8%	-
Cubic	20	Ring (orange)	55%	8.6%	-
Cubic	12	Corners (yellow)	33%	9.4%	-
Cubic	4	Middle (purple)	5.7%	14.5%	15.8%
Cubic	4	Corners (green)	5.7%	12%	16.4%
Cylindrical	4	Middle (red)	7.1%	10.3%	11.6%
Cylindrical	12	Middle (cyan)	21.3%	8.9%	9.4%

## Data Availability

The data presented in this study are available on reasonable request from the corresponding author.
